# Medical Cannabis and Utilization of Nonhospice Palliative Care Services: Complements and Alternatives at End of Life

**DOI:** 10.1093/geroni/igab048

**Published:** 2022-01-14

**Authors:** James A Croker, Julie Bobitt, Kanika Arora, Brian Kaskie

**Affiliations:** 1 Department of Health Management and Policy, University of Iowa, Iowa City, Iowa, USA; 2 Center for Tobacco Control Research and Education, Cardiovascular Research Institute, University of California San Francisco, San Francisco, California, USA; 3 Department of Medicine, University of Illinois at Chicago, Chicago, Illinois, USA

**Keywords:** Pain management, Prescription opioids, Self-reported outcomes, Symptom management

## Abstract

**Background and Objectives:**

There is a need to know more about cannabis use among terminally diagnosed older adults, specifically whether it operates as a complement or alternative to palliative care. The objective is to explore differences among the terminal illness population within the Illinois Medical Cannabis Program (IMCP) by their use of palliative care.

**Research Design and Methods:**

The study uses primary, cross-sectional survey data from 708 terminally diagnosed patients, residing in Illinois, and enrolled in the IMCP. We compared the sample on palliative care utilization through logistic regression models, examined associations between palliative care and self-reported outcome improvements using ordinary least squares regressions, and explored differences in average pain levels using independent *t*-tests.

**Results:**

115 of 708 terminally diagnosed IMCP participants were receiving palliative care. We find increased odds of palliative care utilization for cancer (odds ratio [OR] [*SE*] = 2.15 [0.53], *p* < .01), low psychological well-being (OR [*SE*] = 1.97 [0.58], *p* < .05), medical complexity (OR [*SE*] = 2.05 [0.70], *p* < .05), and prior military service (OR [*SE*] = 2.01 [0.68], *p* < .05). Palliative care utilization is positively associated with improvement ratings for pain (7.52 [3.41], *p* < .05) and ability to manage health outcomes (8.29 [3.61], *p* < .01). Concurrent use of cannabis and opioids is associated with higher pain levels at initiation of cannabis dosing (*p* < .05).

**Discussion and Implications:**

Our results suggest that cannabis is largely an alternative to palliative care for terminal patients. For those in palliative care, it is a therapeutic complement used at higher levels of pain.

**Translational Significance:** Patients using cannabis may delay or refuse palliative care services. Most patients in the Illinois Medical Cannabis Program are using cannabis without any formal supportive comfort care. However, some are using cannabis in addition to palliative care and report using cannabis at higher pain levels. Providers should engage with patients on the potential pros and cons of medical cannabis and palliative care at end of life. Future researchers should examine whether access to cannabis programs further delays utilization of palliative care services by eligible patients.

## Background and Objectives

Medical cannabis use continues to increase among older adults, including those approaching the end of life (EOL) (1–6). For our purposes, EOL patients are those patients who have received certification from their physician of a terminal prognosis of no more than 6 months for their diagnosed conditions. Many EOL patients experience severe, prolonged symptoms stemming from both their condition and their treatments, sometimes extending beyond 6 months, and they are often looking for effective options that can manage their symptoms and increase their comfort (7, 8). Patients have the option to engage supportive care as a means of securing symptom management and accessing a broad range of medical, emotional, social, and spiritual supports (9–11). Although terminally diagnosed patients have historically had the most direct access and broadest options for accessing supportive care, including hospice and nonhospice palliative care services, supportive care at every stage seeks to improve the overall quality of life, efficiently align services, and minimize suffering for patients and their caregivers, with well-established advantages (12–15). Medical cannabis products have also been associated with therapeutic benefits (ie, reduced pain, management of gastrointestinal issues, reduction in sleep disturbances, and mood elevation) relevant to EOL patients (16, 17), and the increase in state cannabis program enrollment among older adults includes terminally diagnosed patients (6, 8).

**Figure 1.**
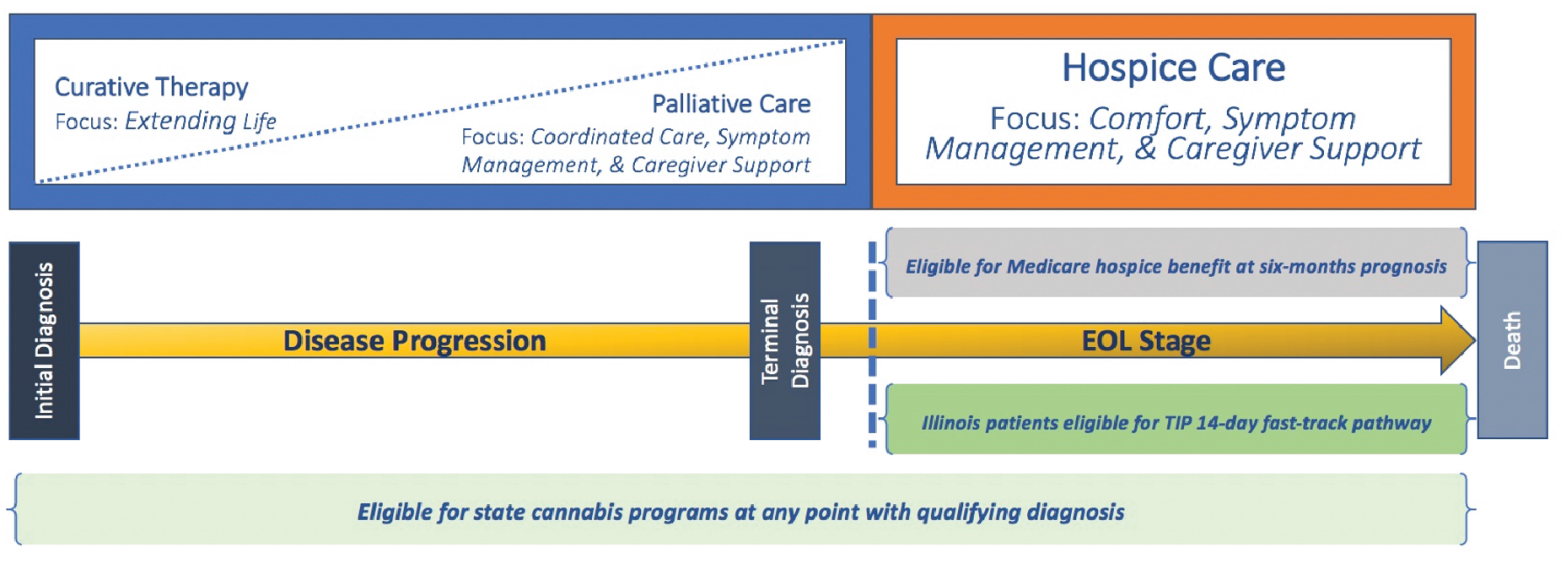
Supportive care continuum for medical cannabis patients near end of life (EOL). Adapted from Refs (18–20).

Supportive care is often framed on a continuum, visualizing when during the course of a patient’s disease various service elements become available (18–20). Supportive care options at EOL include hospice care and palliative care. While these service elements overlap along the supportive care continuum, and the terms are often used interchangeably, there are key differences. Figure 1 presents the supportive care continuum adapted here for medical cannabis patients. The continuum begins with initial diagnosis, follows as the disease progresses, and continues through terminal diagnosis into the EOL stage, that is, the time between terminal diagnosis and death. Starting at initial diagnosis, the patient has access to *standard, curative therapy* where the focus is on treatments, cures, and prolonging life (21). *Palliative care* occurs alongside curative care, usually inside of clinical settings (22). While historically only available as conditions advanced to terminal status, current clinical guidelines call for making palliative care available to patients with any serious condition as close to diagnosis as possible, as early delivery of palliative care services can reduce unnecessary hospitalizations and utilization of costly health services (23, 24). However, according to the World Health Organization, both terminal and nonterminal patients lack sufficient access to quality palliative care services for a variety of reasons, including a general lack of awareness, medical and nonmedical access barriers, internal and external stigma, and other factors that can independently prevent patients from utilizing palliative care (24).

In *hospice care*, the focus shifts to EOL preferences, providing comfort and caregiver supports wherever the patient resides (11). The hospice promise is that patients will have coordinated support throughout the EOL stage, up to and through the moment of death. The Medicare hospice benefit is formally available to patients in the EOL stage once they receive certification of a maximum 6-month prognosis and opt to forgo curative treatments (25).

Medical cannabis, like palliative care, is not limited to terminally diagnosed patients. In the Illinois Medical Cannabis Program (IMCP), it is available to any patient upon diagnosis of a qualifying condition and remains accessible so long as their physician continues to certify their eligibility annually (26). The physician certification requirements were intended to ensure patients were not using cannabis as a therapeutic without some form of medical oversight from a physician familiar with their case. Illinois also created a specific fast-track pathway into the IMCP for EOL patients which is also bound to the certified 6-month prognosis (27). Patients engaging this pathway can apply for medical cannabis as a *complement* (used in addition to) to supportive care. However, it also appears some patients could potentially engage medical cannabis as an outright *alternative* (used in place of) to supportive care and miss out on the range of benefits these services provide.

What role does access to medical cannabis play in observed variations in palliative care utilization? For some EOL patients, cannabis may be operating as a complement to palliative care, where they receive cannabis for symptom management in addition to coordinated support services and curative treatments for their primary condition, if they so desire (11, 19). However, many patients at EOL are not engaged in any form of supportive care. There is a need to know more about the use of cannabis among terminally diagnosed older adults, and whether it operates as a *complement* or *alternative* to nonhospice palliative care.

### Medical Cannabis: A Complement or Alternative to Palliative Care?

For many in the EOL sector, cannabis is already viewed as a form of complementary medicine for symptom management. Cannabis use in palliative care emerged with concerns around potentially excessive and long-term use of prescription opioid medications (28–31). In particular, EOL care providers were concerned about opioid-induced constipation (OIC) and other gastrointestinal issues, along with potential opioid misuse, dependence, and accidental overdose. Unlike hospice care, which is relatively less concerned with the potential harms of extended opioid use, palliative care aims at reducing suffering without violating the ethical principle of “nonmalfeasance” as defined by state regulations (32). In some cases, palliative care has been associated with greater longevity when initiated earlier in the course of care (29, 30). The importance of drug safety, therefore, remains preeminent in nonhospice palliative care, and providers must deal with the side effects of high doses and the negative effects of pill burden on patients (28, 31).

While we do not know the extent to which people use cannabis as a complement to palliative care, we do know medical cannabis use is associated with several desirable outcomes, specifically the management of various symptoms, including chronic pain, chemotherapy-induced nausea, AIDS-related cachexia, and multiple sclerosis spasticity (16, 17, 33–36). The use of cannabis as a complement might also reduce potential risks from extended opioid use and polypharmacy including OIC, nausea, vomiting, respiratory depression, and potential dependence (28, 37–40). Many patients in palliative care also express positive attitudes about cannabis and its therapeutic potential, regardless of whether or not they reside in a state permitting its use or their outpatient care setting (41, 42).

Variation and disparities in access and utilization of palliative care services raise concerns about both quality of care and equity for EOL patients. Demographic groups that are less likely to engage in palliation include males, married individuals, and those with low socioeconomic status (43–46). Differences in palliative care utilization by gender have been attributed to the greater likelihood for women to engage in health care services and to receive “less aggressive” care when they do (45). Differences in spousal education levels and informal caregiving roles and expectations may also lower nonhospice palliative care utilization by married men (46). Males and unmarried individuals are also more likely to engage in cannabis use (1, 2, 47, 48). Palliative care utilization also varies by system-level access to care measures like health insurance coverage and access to health care through the Veterans Administration (VA) (49, 50).

It is also possible that cannabis use could operate more as an alternative for some EOL patients, where cannabis is used as an alternative to prescription opioids and other approaches for pain management (51), and patients forgo the advantages of medical case management and wrap-around services available to them in palliative care. Do palliative care patients using cannabis concurrently with opioid medications see better management of pain than patients using cannabis as an alternative? Unfortunately, we lack sufficient evidence to answer this question.

The objective of this study is to explore differences among the terminal illness population within the IMCP by their use of palliative care services. This study will expand upon previous research on terminal patients in the program by identifying key predictors for palliative care utilization, along with associations between palliative care utilization and health outcomes, and the concurrent use of cannabis and prescription opioids for pain management. Specifically, we seek to answer 3 questions. First, what characterizes the terminal patients in the sample using palliative care from those terminal patients engaging only standard care? Second, do we see evidence of medical cannabis operating as an effective complement or alternative for symptom management among terminally diagnosed older adults using nonhospice palliative care? And third, are palliative care patients using cannabis at lower pain thresholds when using cannabis concurrently with prescription opioids, than when using cannabis alone for pain management?

To answer these questions, we use a previously developed framework for understanding EOL care planning to explore decision making among this population between palliative care and standard treatment without supportive care (6). We hypothesize (a) terminal palliative care patients will differ from terminal patients in standard care with generally lower health status, and greater condition complexity, but will experience fewer access barriers to the IMCP; (b) palliative care will be positively associated with improvements to gastrointestinal issues, pain, and quality of life measures; and (c) palliative care patients using opioids will report initiating cannabis dosing at higher pain intensity and will report lower average 30-day pain scores than palliative care patients not using opioids.

## Research Design and Methods

Using cross-sectional data from a survey targeting older adults using medical cannabis in Illinois, we explore the decision by terminal patients on whether to engage palliative care services for EOL care, their differences on a range of self-reported outcomes, and how the use of cannabis as a complement or alternative to EOL care affects their use of opioids and severity of pain symptoms. The data come from a previously tested, anonymous, closed access email survey of adults who enrolled in the IMCP prior to October 31, 2019. The instrument went through multiple rounds of testing in the development stage, including pilot testing in samples from the Illinois and Colorado medical cannabis programs (4–6, 51).

Eligible participants were contacted with assistance from Illinois Department of Public Health (IDPH). A link to the survey was emailed to 17 405 unique email addresses associated with enrolled patients, of these 821 were undeliverable, leaving an eligible *n* of 16 584. Reminder requests were emailed 2 days, 30 days, 6 weeks, and 8 weeks after the initial request. Participants were invited to connect to the online survey via REDCap (Research Electronic Data Capture). The survey was available to complete between October 31 and December 31, 2019. Participants were informed of the research purpose, completion time, data storage protocols, and contact information for the study personnel and provided consent prior to completing the survey. Approval for this research was granted by the Internal Review Board at the University of Illinois at Urbana Champaign. No incentives were offered for participation. Cookies were not used for identification; however, duplicate entries were identified by self-reported email addresses and eliminated before analysis, keeping the more complete entry. In total, 4 066 unique responses were collected from eligible participants. The overall survey response rate was 24.5% (4 066/16 584) among all IMCP enrollees. According to 2019 IDPH reports, there were a total of 891 terminal illness patients enrolled in the program (27). Within our sample, 727 respondents indicated that they were terminal patients, producing a response rate of 82% (727/891) among all terminal IMCP patients.

### Sample

**Figure 2.**
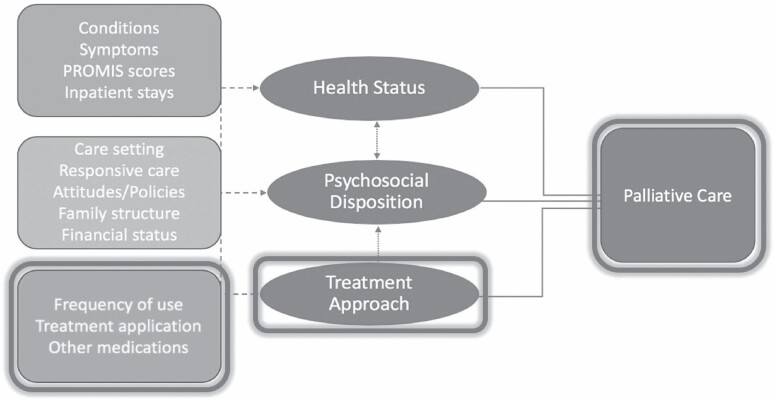
Palliative care decision framework for cannabis patients near end of life.

The target population for the survey was adults aged 60 years and older enrolled in the Illinois cannabis program. However, given some respondents were proxy responses for program participants, a small number of respondents (*n* = 9) indicated age younger than 60 years. To be included in the EOL analytic sample, a respondent had to indicate receiving a terminal condition diagnosis with a maximum 6-month prognosis or report completing the application for the IMCP via the 14-day terminal illness “fast-track” application, and to be included in this study, they also could not indicate being enrolled in hospice care. In total, 708/727 EOL respondents were included in the analyses. Of these, 115 (16%) reported receiving nonhospice palliative care services. The other 593 (84%) terminal patients were pursuing standard care. The full analytic sample structure is shown in Figure 2.

### Data

The survey included adaptive questioning measures related to health status, cannabis use, and experiences with palliative care along with sociodemographic characteristics. The full survey questionnaire is included in Supplementary Material A.

### Independent Variables

**Figure 3.**
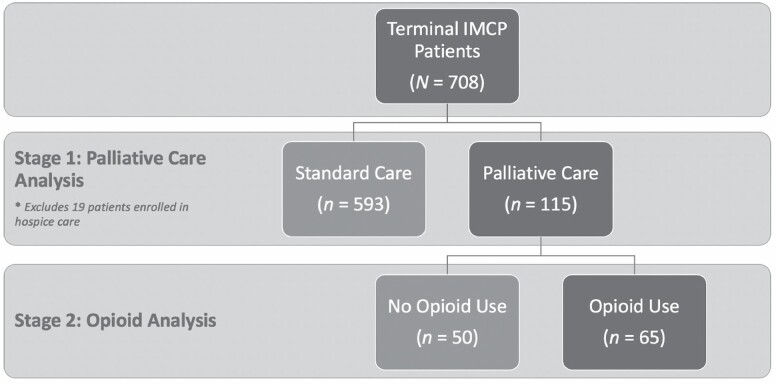
Analytic sample structure (*n* = 708). IMCP = Illinois Medical Cannabis Program.

Figure 3 shows the conceptual framework for EOL care decision making and the corresponding item measures pulled for analysis. The conceptual model was developed a priori to the study’s design and data collection phase, as a result of exploratory analysis and an extensive literature review on the factors driving utilization of palliative care. In our view, the decision to engage palliative care services by terminally diagnosed patients is shaped by 3 patient-level factors:

(1) Health status: the health status factor includes measures for certifying condition, symptoms, medical complexity, inpatient hospital stays, and ability to manage chronic conditions.
(2) Psychosocial disposition: the psychosocial disposition factor includes established desired hospice setting, desire for responsive care, trust in health care providers, in addition to measures for physician attitudes, institutional cannabis policies, marital status, financial security, and caregiver proxy use.
(3) Cannabis access and treatment approach: the cannabis treatment factor captures measures relating to health care and treatment access including insurance coverage of certification visit, type of physician certifying patient eligibility, and relationship with the physician. We also include measures capturing medical cannabis use behaviors, specifically, indicators for treatment purpose, frequency of use, and dosing methods.

In this exploratory analysis, we examine how these factors relate to a terminal patient’s EOL care decision making.

Demographic variables used in all analyses included dichotomous indicators constructed for age group category, gender, race/ethnicity, marital status, educational attainment, prior military service, and financial status. A dichotomous indicator is also included for caregiver proxy use. Illinois allows cannabis patients to certify a caregiver who is able to purchase and possess the cannabis on behalf of the patient. Caregiver proxies were therefore identified given the possibility they may be the contact email address on file with IDPH. Caregiver proxies are also often used in EOL studies to accommodate patients who may have more advanced disease states or who may face recall issues (52–54). When assessing quality and satisfaction, proxies have been observed to have higher quality/satisfaction scores while also having more negative reports on clinical nursing and care coordination (55–57). The research shows the difference is small, but statistically significant. Caregiver proxy use was originally included in the psychosocial disposition factor when the conceptual model was initially designed for hospice patients, because of the unique role and influence informal caregivers have on the hospice enrollment decision process (6). The authors debated shifting the placement of caregiver proxy use to the health status factor because of the potential for greater proxy use among patients with more advanced illness or moving it into the cannabis use factor because all IMCP patients were able to certify a caregiver who could access and manage cannabis products (and potentially answer the questionnaire) on their behalf, regardless of a patient’s disease state. Because of this potential to rest in any of the 3 factors, we chose not to change its placement for this study.

Self-reported health conditions qualifying patients for the cannabis program were grouped into dichotomous indicator variables for analysis including cancer, mental health disorders, musculoskeletal disorders, neurological disorders, other terminal illnesses, multiple morbidity, and medically complex cases (defined here as 3 or more chronic conditions, AIDS complications, and a terminal diagnosis). Symptoms treated with cannabis included pain, difficulty sleeping, emotional problems, gastrointestinal issues, and multiple co-occurring symptoms. Pain status was assessed using an 11-point pain scale (0–10, where 0 = “No Pain,” 1–3 = “Mild Pain,” 4–6 = “Moderate Pain,” 7–9 = “Severe Pain,” 10 = “Worst Possible Pain”) (58). Global measures for capturing self-reported assessments of physical health status, mental health status, and ability to manage health status were also included. Categorical health status responses (1 = “poor,” 2 = “fair,” 3 = “good,” 4 = “very good,” and 5 = “excellent”) and symptom frequency ratings (1 = “almost always,” 2 = “often,” 3 = “sometimes,” 4 = “rarely,” and 5 = “never”) were transformed into dichotomous indicators capturing reports of lower health status and greater symptom frequency for analysis (“low health status” or “frequent symptoms”—“no” = 3, 4, and 5; “yes” = 1 and 2).

Cannabis use was assessed with measures capturing purpose (medical only/combined medical and recreational), frequency of use in past 30 days, dosing methods (smoke inhalation, vaporizer, edible products, oral pill/tablet, cream/ointment), status as a new or “naïve” cannabis user in later life, and reports of negative experiences with cannabis use in the past year. To identify potential barriers to accessing the cannabis program, we contrasted outcomes by the source of patient knowledge about the IMCP, whether or not their certifying physician was a routine provider, whether or not they entered the IMCP through the fast-track application, and whether or not the patient’s health insurance covered the cannabis certification visit with their doctor.

### Outcome Measures

Outcomes for this analysis focus on self-reported improvements to health status measures for gastrointestinal issues, sleep quality, emotional issues, ability to manage health status, and quality of life. Patients were asked “How does cannabis affect your [health outcome measure],” with 3 categorical response options (“Makes it worse,” “No Change,” and “Makes it better”).

Patients who indicated cannabis use had a positive or negative effect on their general health or specific symptoms were invited to provide a continuous impact rating score via a drag-bar scale (0–100). Outcome measures for the opioid analysis include average pain levels at the initiation of cannabis use and average pain levels over the past 30 days.

### Statistical Analyses

We engage a 3-stage exploratory approach to test a range of variables based on the decision framework we developed for this study. Stage 1 descriptive statistics included palliative care utilization versus nonpalliative care utilization (usual care) among terminally diagnosed patients, cannabis and other substance use behaviors, motivations for medical cannabis use, palliative care experiences, along with other variables included based on our decision framework. We perform univariate analyses to describe the sample and identify variables with strong associations to include in the regression models. Respondent count and proportion were calculated based on total respondents per question, missing observations were treated as missing at random and the number skipping a question was not included in the denominator. To determine group differences for continuous variables (age in years, pain levels, 30-day use frequency), we compare the item means using independent sample *t*-tests. To determine group differences for discrete variables (age group, gender, education, marital status, caregiver proxy use, etc.), we use chi-square tests. A *p* value of .05 or less on a 2-tailed test was considered statistically significant.

In Stage 2, we use a logistic regression model to compare those terminal patients engaged in palliative care to those not pursuing palliation in order to examine the significant correlates of palliative care utilization among terminal IMCP patients. Independent variables in the models included dichotomous indicators for age groups (1 = *under age 65 years*, 2 = *age 65–69 years*, 3 = *age 70–79 years*, and 4 = *age 80 years and older*), gender (male/female), education (less than college/college degree or more), marital status (not married/married), prior military service (nonveteran/veteran), financial security status (secure/insecure), and caregiver proxy use (no/yes), in addition to those variables shown in the Stage 1 analysis to have statistically significant associations. As a robustness check, we calculate propensity scores using psmatch2 in Stata, where the outcome of interest is the patient’s reported reason for using cannabis (ie, “I use [cannabis] as part of palliative care”) and included the items from the Stage 1 analysis as predictor variables. We then run an additional logistic regression model that includes the propensity score as a covariate.

Finally, in Stage 3, our exploratory approach turns to self-reported outcomes. In order to explore differences in health outcomes, we first use a series of ordinary least squares (OLS) regression models to identify incremental associations to self-reported improvement scores for palliative care, with combined cannabis and opioid use, frequency of use, and medical complexity included as independent variables. The linear regressions take the general form:


$$\begin{array}{ll} Y_{i}= & \;\beta_{0i}+\;\beta_{1i}pc+\;\beta_{2i}opioids+\;\beta_{3i}frequency\\ & +\;\beta_{4i}complex+\;\beta_{5i}demos+\varepsilon_{i} \end{array}$$

where Y represents the improvement score [0–100] for the outcome measure (quality of life, ability to manage health status, pain, sleep quality, emotional problems, and gastrointestinal issues), *pc* is an indicator for palliative care utilization, *opioids* is an indicator for the complementary use of cannabis with prescription opioids, *frequency* captures 30-day cannabis use frequency in days, *complex* captures medical complexity associated with their diagnosed conditions, and *demos* captures the demographic measures. Table 1 presents the models and items included in the 3-stage exploratory approach.

**Table 1.** Analytic Variables Included in the 3-Stage Modeling Approach

Model	Demographic Measures	Health Status Measures	Cannabis Use Measures	Program Access Measures
Univariate analyses and tests of statistical significance	• Age in years • Age 80 years and older • Sex • Race • College education • Marital status • Employment status • Financial insecurity	• Caregiver proxy • Disability status *Global measures* • Low QOL • Difficulty managing health outcomes • Low psychological well-being • 30-day pain levels • Emotional problems • Frequent GI issues *Qualifying condition* • Cancer diagnosis • Another illness *Condition severity* • Multiple conditions • Medically complex *Symptoms* • Pain • Emotional issues • GI issues • Multiple symptoms • Past-year opioid use	*Cannabis use purpose* • Medical use only • Combined recreational use • 30-day cannabis use frequency *Cannabis dosing method* • Smoke inhalation • Oral pill/tablet • Edibles • Naïve users • Past-year negative cannabis experience	• Certified by a routine provider • TIP fast-track applicant • Health insurance • Military veteran
Logistic regression model*	• Age 80 years and older • Sex • College education • Marital status • Military veteran • Financial insecurity	• Caregiver proxy • Low QOL • Low psychological well-being • Frequent GI issues • Multiple symptoms • Opioid use • Propensity score*		• TIP fast-track applicant • Health insurance
Linear regression models		*Outcomes*: • QOL • Ability to manage outcomes • Psychological well-being • Pain • Emotional problems • GI issues	*Independent variables:* • Palliative care • Combined cannabis and opioid use in the past year • 30-day cannabis use • Cancer diagnosis • Demographics • Caregiver proxy use	

*Notes:* QOL = quality of life; GI = gastrointestinal; TIP = terminal illness program.

*A second logistic regression that included the propensity score as a covariate was included in the second stage as a robustness check.

To examine associations between the concurrent use of cannabis and prescription opioids and pain management among patients utilizing palliative care services, we use independent *t*-tests to assess differences for average pain levels at initiation of cannabis dosing and average 30-day pain levels.

Some measures do have reduced *n*s by virtue of the adaptive questionnaire structure. In particular, this is the case in the OLS regression models, where the *n*s for the individual outcome measures vary because the response was restricted to capturing only patients who reported using cannabis to specifically treat the corresponding symptoms. Overall, the missing data are primarily on demographic measures (specifically age, gender, race/ethnicity, military service, and financial status). Missing data for all independent variables included in the analyses are below 5% and are considered missing completely at random. Our threshold of significance was a *p* value of .05 or less being considered statistically significant on all 2-tailed tests. All data management and statistical analyses were performed using Stata 16.1 (StataCorp LLC, College Station, TX). Reporting here is consistent with the CHERRIES and STROBE checklists for cross-sectional studies (59, 60).

## Results

Table 2 presents descriptive measures for cannabis use behaviors, reasons for use, and palliative care experiences status for all terminal patients in the sample. In total, 115 (16%) of 708 terminal IMCP patients indicated that they were currently engaged in nonhospice palliative care. Terminally diagnosed patients in the IMCP range in age from 60 to 91 (as noted, 9 caregiver proxy respondents in the sample providing age data were younger than age 60). Caregiver proxies are used by 8% of the terminally diagnosed patients in standard care and 18% of the respondents in nonhospice palliative care.

**Table 2.** Cannabis Use Behaviors and Non-Hospice Palliative Care Patient Experience Measures (*N* = 708)

Cannabis and substance use behaviors	Standard Care Patients (*n* = 593)		Palliative Care Patients (*n* = 115)	
	Obs.	%	Obs.	%
*Average monthly spending on cannabis*				
Between $1 and $99	203	0.34	36	0.31
Between $100 and $199	190	0.32	45	0.39
Between $200 and $299	94	0.16	13	0.11
Between $300 and $399	47	0.08	9	0.08
More than $400	52	0.09	12	0.10
*Cannabis dosing method*				
Smoke inhalation	258	0.44	44	0.38
Vaporizer	216	0.36	43	0.37
Oral pill/tablet	131	0.22	27	0.23
Liquid tincture	176	0.30	50	0.43
Edible product	365	0.62	65	0.57
Cream/ointment	217	0.36	33	0.29
*Cannabis use in the past year*				
A few times	53	0.09	12	0.10
1-4 times per month	48	0.08	11	0.10
Once or twice per week	55	0.09	4	0.03
Regularly (3 or more times per week)	153	0.26	29	0.25
Daily (1 or more times per day)	282	0.48	59	0.51
*Reasons for using medical cannabis*				
Prescribed medications do not help enough	323	0.48	40	0.35
I prefer not to take prescription medication at all	218	0.32	21	0.18
I prefer not to take these prescription medications more than necessary	291	0.43	44	0.38
My primary doctor or specialist said cannabis would help	227	0.33	27	0.23
Nonprescription treatments (eg, physical therapy, counseling) do not help enough	176	0.26	19	0.17
I use as palliative care	85	0.13	42	0.37
*Before you began the state program, had you been using cannabis for a medical purpose?*				
No	454	0.77	97	0.84
Yes	139	0.23	18	0.16
Naïve/new cannabis user at program enrollment	255	0.43	55	0.48
Had negative experience with cannabis use in the past year	74	0.12	14	0.17
*Prescription medication use*				
Opioid use	269	0.45	65	0.57
Benzodiazepine use	199	0.34	47	0.41
*Palliative care experiences*				
Using cannabis specifically as part of your palliative care			94	0.82
Palliative care provider approves of cannabis use (*n* = 94)			91	0.97
Provider’s attitude was the reason why provider was selected (*n* = 94)			33	0.35
Pain level at cannabis use initiation (*0*–10), *n* = 94, mean			92	5.57

Table 3 presents the univariate analyses and comparisons of means by palliative care utilization for items included in Stage 1 based on the conceptual framework. Table 4 presents the results of logistic regression comparing terminal patients in palliative care to those not in palliative care. When controlling for demographic variables we find 2 times greater odds of palliative care utilization for military veterans (odds ratio [OR] [*SE*] = 2.01 [0.68], *p* < .05) in the sample. However, we find 50% lower odds of palliative care utilization for college graduates (OR [*SE*] = 0.50 [0.12], *p* < .001), 38% lower odds for those who are married (OR [*SE*] = 0.62 [0.15], *p* < .05), and 47% lower odds for those in the sample not experiencing financial insecurity (OR [*SE*] *=* 0.53 [0.13], *p* < .01). In terms of health status measures, we observed over 2 times the odds of palliative care use for those terminal patients in our sample diagnosed with cancer (OR [*SE*] = 2.15 [0.53], *p <* .01), and those reporting medical complexity (OR [*SE*] = 2.05 [0.70], *p* < .05). We observed 97% greater odds of palliative care utilization for patients reporting low psychological well-being (OR [*SE*] = 1.97 [0.58], *p* < .05), and 75% greater odds for those using cannabis to treat gastrointestinal issues (OR [*SE*] *=* 1.75, *p* < .05). However, for cannabis use and access measures, we observed 53% lower odds of palliative care use for those patients who used the fast-track application into the IMCP (*OR* [*SE*] *=* 0.47 [0.11], *p* < .001), and 67% lower odds for those whose cannabis certification visits were covered by their insurance (OR [*SE*] = 0.33 [0.08], *p* < .001).

**Table 3.** Univariate Analyses With Means Comparisons and Tests of Significance for Terminal Patients by Nonhospice Palliative Care Utilization Status (*N* = 708)

	Standard Care Mean (*SE*) (*n* = 593)	Palliative Care Mean (*SE*) (*n* = 115)	p
*Demographic measures*			
Age in years (range: 34–91 years)	67.17 (0.28)	67.54 (0.69)	.71
Younger than age 65 years, % (*SE*)	0.37 (0.02)	0.42 (0.05)	.20
**Age 65–69 years, % (*SE*)**	**0.35 (0.02)**	**0.24 (0.04)**	**.04**
Age 70–79 years, % (*SE*)	0.24 (0.02)	0.27 (0.05)	.92
80 years or older, % (*SE*)	0.04 (0.01)	0.07 (0.03)	.24
Females	0.53 (0.02)	0.54 (0.05)	.86
Non-White	0.07 (0.01)	0.09 (0.03)	.74
College degree or more	0.47 (0.02)	0.41 (0.05)	.34
Married	0.63 (0.02)	0.57 (0.05)	.20
Prior military service	0.12 (0.02)	0.20 (0.04)	.06
Presently employed	0.23 (0.02)	0.20 (0.04)	.39
**Financially secure**	**0.73 (0.02)**	**0.66 (0.05)**	**.04**
*Health status measures*			
**Caregiver proxy use**	**0.08 (0.01)**	**0.18 (0.04)**	**.01**
Disabled	0.40 (0.02)	0.50 (0.05)	.09
**Low quality of life**	**0.35 (0.02)**	**0.46 (0.05)**	**.01**
Difficulty managing outcomes	0.21 (0.02)	0.26 (0.05)	.32
**Low psychological well-being**	**0.18 (0.02)**	**0.29 (0.05)**	**.01**
30-day pain levels (0–10)	5.21 (0.11)	5.00 (0.27)	.63
Frequent emotional issues	0.24 (0.02)	0.30 (0.05)	.32
**Frequent gastrointestinal issues**	**0.20 (0.02)**	**0.30 (0.05)**	**.01**
**Cancer diagnosis**	**0.34 (0.02)**	**0.65 (0.05)**	**<.001**
**Noncancer terminal diagnosis**	**0.07 (0.01)**	**0.18 (0.04)**	**<.001**
Multiple diagnoses	0.22 (0.02)	0.20 (0.04)	.25
**Medically complex**	**0.08 (0.01)**	**0.20 (0.04)**	**<.001**
Treating pain symptoms	0.83 (0.02)	0.78 (0.04)	.19
Treating emotional problems	0.39 (0.02)	0.46 (0.05)	.13
**Treating gastrointestinal issues**	**0.26 (0.02)**	**0.46 (0.05)**	**<.001**
**Treating multiple symptoms**	**0.72 (0.02)**	**0.80 (0.04)**	**.05**
**Opioid use in the past year**	**0.45 (0.02)**	**0.57 (0.05)**	**.03**
*Cannabis use and program access measures*			
Medical use only	0.82 (0.02)	0.88 (0.03)	.27
Both recreational and medical	0.21 (0.02)	0.14 (0.04)	.50
Days using cannabis (0–30)	21.36 (0.47)	19.29 (1.18)	.13
Smoke inhalation	0.45 (0.02)	0.38 (0.05)	.30
Oral pill/tablet	0.22 (0.02)	0.24 (0.04)	.74
Naïve (first-time) user	0.33 (0.02)	0.35 (0.05)	.54
**Fast-track application**	**0.80 (0.02)**	**0.65 (0.05)**	**<.001**
**Insurance coverage**	**0.71 (0.02)**	**0.58 (0.05)**	**.03**
Negative experience	0.12 (0.01)	0.17 (0.04)	.64

*Notes:* Items in bold were observed to have statistically significant differences between groups (*p* ≤ .05). These items were pulled for inclusion in the logistic regression analysis.

**Table 4.** Logistic Regression Predicting Palliative Care Utilization: Comparing Terminal Patients in Palliative Care to Terminal Patients Not Engaging Supportive Care (*n* = 633)

Palliative Care Patients	OR	95% CI	p
*Demographics*			
College degree or more	0.50	0.31–0.80	<.001
Married	0.62	0.39–0.99	.05
Prior military service	2.01	1.03–3.90	.04
Not experiencing financial insecurity	0.53	0.33–0.87	.01
*Health status*			
Low psychological well-being	1.97	1.11–3.51	.02
Cancer diagnosis	2.15	1.32–3.49	<.001
Medically complex	2.05	1.05–3.99	.03
Treating gastrointestinal issues	1.75	1.02–3.00	.04
*Cannabis use and program access*			
14-day fast-track applicant	0.47	0.30–0.75	<.001
Insurance covered certification	0.33	0.21–0.52	<.001

*Notes:* OR = odds ratio; CI = confidence interval. This logistic regression included indicators for age group category, gender, race/ethnicity, employment status, caregiver proxy use, low quality of life, frequent gastrointestinal issues, multiple symptoms, opioid use in the past year as covariates.

As a robustness check, we engaged propensity score matching and found high levels of common support. When conducting logistic regression that included the propensity score as a covariate, we observed significantly greater odds of nonhospice palliative care utilization for terminal patients in the program with a cancer diagnosis and lower odds of utilization for college-educated and married individuals in our sample. While such an approach is a common practice, it should be noted the inclusion of the propensity score inside of the regression model is often associated with bias in the treatment effect estimate, usually toward the null (61–63). The results of this secondary analysis are presented in Supplementary Table 1, Supplementary Material B.

When engaging OLS regression modeling of the measures capturing symptom changes from cannabis use, we find palliative care utilization is significantly associated with higher improvement ratings for pain (β [*SE*] = 7.52 [3.41], *p* < .05) and ability to manage health outcomes (β [*SE*] = 8.29 [3.61], *p* < .01). Concurrent use of cannabis and prescription opioids was associated with ability to manage health outcomes (β [*SE*] *=* 6.28 [2.50], *p* < .01) and with health-related quality of life (β [*SE*] = 4.49 [2.28], *p* < .05). We also find cannabis use frequency had consistent positive associations with beta coefficients ranging from 1.61 (0.11) to 1.92 (0.12), *p* < .001. The results of the OLS regressions are presented in Table 5.

**Table 5.** OLS Regression Beta Coefficients of Self-Reported Improvements to Health Outcomes

	GI	Pain	Sleep	EMO	MNG	PSY	QOL
	Coeff. β (*SE*) (*n* = 211)	Coeff. β (*SE*) (*n* = 435)	Coeff. β (*SE*) (*n* = 406)	Coeff. β (*SE*) (*n* = 306)	Coeff. β (*SE*) (*n* = 451)	Coeff. β (*SE*) (*n* = 355)	Coeff. β (*SE*) (*n* = 488)
Palliative care	1.75 (4.95)	7.52* (3.41)	5.04 (3.58)	3.93 (4.02)	8.29** (3.61)	3.43(3.97)	4.27 (3.17)
Days using cannabis (0–30 days)	1.65*** (.16)	1.61*** (.11)	1.69*** (.10)	1.92*** (.12)	1.69*** (.11)	1.92*** (.13)	1.79*** (.097)
Opioid use in the past year	–0.58 (3.96)	3.84 (2.45)	4.26 (2.48)	4.03 (2.93)	6.47** (2.49)	3.73 (2.95)	4.49* (2.28)
Cancer diagnosis	9.36* (4.05)	4.63 (2.63)	8.49*** (2.62)	8.27** (3.01)	6.64* (2.71)	6.90* (3.08)	6.26* (2.44)
Medically complex	–5.94 (6.23)	2.69 (4.24)	–7.09 (4.37)	–11.49* (4.80)	–3.59 (4.33)	–7.81 (4.82)	–4.41 (4.00)

*Notes:* OLS = ordinary least squares; GI = gastrointestinal issues; EMO = emotional issues; MNG = ability to manage health outcomes; PSY = psychological well-being; QOL = health-related quality of life. OLS regressions included demographic covariates (ie, age category, gender, race/ethnicity, marital status, educational attainment, prior military service, financial security status) along with an indicator for caregiver proxy.

*p < .05, **p < .01, ***p < .001.

### Concurrent Cannabis and Opioid Use for Pain Management

In total, 65 (57%) of 115 terminal patients in palliative care reported using opioids in the past year. Table 6 presents the results of independent *t*-tests examining the differences in average pain levels for palliative care patients by opioid use in the past year. Patients in palliative care who were using cannabis concurrently with opioids had significantly higher pain levels at initiation of cannabis dosing (mean difference [*SE*] *=* −1.12 [0.56], *t =* −2.00, *p* < .05) than those not using prescription opioids.

**Table 6.** Independent *t*-Tests Comparing Differences in Average Pain Levels Among Nonhospice Palliative Care Patients by Concurrent Use of Opioids in the Past Year (*n* = 115)

	Opioid nonusers (*n* = 50) Mean (*SE*)	Opioid users (*n* = 65) Mean (*SE*)	Difference Mean (*SE*)	*t*-Score	*p*
Pain level at initiation of cannabis dosing	4.92 (.47)	6.04 (.30)	−1.12 (.56)	−2.08	.04
Average 30-day pain level	4.57 (.40)	5.55 (.32)	−0.98 (.51)	−1.88	.05

*Note:* Pain levels (0–10, where 0 = “No Pain,” 1–3 = “Mild Pain,” 4–6 = “Moderate Pain,” 7–9 = “Severe Pain,” 10 = “Worst Possible Pain”).

## Discussion and Implications

This study gives greater insight into the relationship between palliative care and cannabis use among patients near EOL. It indicates that the terminally diagnosed patients in our convenience sample using palliative care are statistically different on demographic, health status, and cannabis use access measures from those terminal patients not engaging in any form of supportive comfort care. It also suggests that terminal palliative care patients in our sample are pursuing a more therapeutic approach, reporting medical purpose only use, complementary use to enhance prescription medications, naïve/first-time cannabis use, and use on physician recommendation. Most patients in our sample are not engaging in formal palliative care services, and a large share of patients utilizing palliative care were not using prescription opioids.

We find support for our first hypothesis that palliative care patients have significant differences from patients in usual care driven by health status, greater condition complexity, and nonmedical access barriers shaping their EOL care choices. Something we did not expect was statistical difference between the groups in our sample coming from predisposing demographic measures. The positive association with prior military service was not anticipated but is understandable as a system effect coming from VA. The VA has repeatedly emphasized quality and cost benefits shown in palliative care as a benefit for their population (47). These results seemingly affirm the efforts by physicians in the VA system to provide access to palliative care for Veterans with terminal diagnoses. Moreover, the IMCP has specific access pathways for Veterans. These results suggest that this targeting approach is having some positive impact on our sample.

Also surprising were the negative associations with financial, education, and marital status. The negative associations for education and marital status were observed in the logistic regression model with the propensity score included. Given the literature demonstrating individuals with lower socioeconomic status are less likely to be offered palliative care services and more likely to be treated with aggressive disease-based care (64–66), we anticipated financial barriers to care would limit access to palliative care services for those experiencing financial insecurity, the opposite was true in this sample. We did not anticipate that higher education or being married would be negatively associated with palliation. Higher education has historically been associated with greater odds of palliative care utilization. Differences by marital status alone have generally not held significant differences (65, 67). One explanation could be attributable to the role of psychological well-being. A person obtaining adequate psychosocial support from a spouse could have less need for the type of emotional support often included in palliative care services. Still, the bundle of services offered as part of palliative care specifically incorporates support for family and informal caregivers, so this finding warrants future investigation.

Finally, we find evidence palliative care patients in this sample are less likely to access the IMCP through the state’s fast-track application and less likely to have their certification visit covered by health insurance. Much like previous findings from hospice patients in the program (6), palliative care patients seem to be entering the IMCP outside of the specialized terminal illness pathway created for them.

We find some support of our second hypothesis that palliative care is positively associated with improvements on a range of outcomes, with consistent significant associations observed for complementary cannabis in palliation on key outcomes, both proximal and distal to the patient, though we expected to see consistent positive associations across the range. While it is impossible to express a causal relationship with this data, these findings combined with consistent positive associations for cannabis use frequency on all measures, and positive association between concurrent opioid use and pain improvement, offer support for positive associations for cannabis use and outcomes pertinent to palliative care patients.

Despite a large share of terminal patients (both palliative care and nonpalliative care) reporting not using opioids in the past year (*n* = 369, 52%), we find partial support for our third hypothesis that cannabis use for symptom management occurs at higher levels for palliative care patients using opioids. However, we do not see this when we ask patients to recall their average pain severity over the past 30 days. Given the large proportion of all survey respondents who reported no prescription opioid use in the past year while also using medical cannabis, the question emerges whether cannabis has acted as a historic substitute to avoid prescription opioid use. Certainly, many patients in the Illinois program are newly using medical cannabis as a substitute through the state’s opioid alternative pilot program, but that population was not sampled as part of this study (51). Moreover, a historic supplementation effect does not seem to be the case when we look at other measures capturing substance use behaviors. Only 157 (22%) of respondents indicated using “medical cannabis” before entering the program, and 310 (44%) of all patients in the sample were completely new to cannabis use on joining the program. Given the lack of longitudinal observation, the data prevent us from demonstrating any causal relationship; however, the associations observed here combined with previous findings on the potential for cannabis as an opioid alternative suggests opportunities for further research on the complex relationship between palliation, opioids, and concurrent cannabis use in more representative samples.

### Limitations

The study has several limitations that should be considered. The primary limitation affecting our results comes from the evidence of selection, nonresponse, and social desirability bias, particularly within the EOL sample population. While these types of biases are common in studies asking questions related to substance use behaviors, they are also particularly challenging for questions about EOL. Selection, in particular, is a challenge that must be acknowledged, as we had no means of controlling for who in the sample were or were not receiving palliative care or using prescription opioids. Due to the structure of the data, we are also limited in terms of what we can know about the patients engaged in palliative care, the availability of palliative care services in their geographic area, and the nature of the care they have experienced. We were unable to link to patient-level data that would inform exactly where they were in the course of their disease, or if the patient has died since answering the questionnaire. Still, the study offers rich exploratory data not otherwise available. Another limitation is the low survey response rate among all respondents. However, the study finds strength in the sizable sample generated among patients in the terminal illness population. Despite the limitations stemming from the study design and the nature of the data, our work helps lay the foundation for more empirical research on the question of medical cannabis in palliative care by identifying associations observed among this convenience sample that can help structure new hypotheses for testing in larger, nationally representative data sets.

### Translational Implications

This study examined the intersection of medical cannabis and palliative care among terminally diagnosed older adults in the IMCP. The findings indicate the majority of terminally diagnosed patients in the program are using cannabis without any formal supportive comfort care, but some are using cannabis in addition to palliative care and using it at higher levels of pain. But, as with other patients who would benefit but are not engaged in palliative care, EOL patients in the IMCP may be avoiding these services because of fear, stigma, access barriers, or other issues independent of their cannabis use. The results offer rich results for further inquiry into cannabis for patients near EOL. Future researchers should frame studies to examine the clinical value of cannabis as a complement to palliative care, and whether participation in state cannabis programs can operate as a potential barrier to supportive care services available across the continuum. The results also offer immediate implications for clinicians, social workers, and other providers. Health care providers should be willing to initiate and engage in discussion with patients on the potential pros and cons of medical cannabis and palliative care at EOL. Where possible, health systems should ensure the availability of provider education and trainings on both medical cannabis and EOL, given many patients look to providers for guidance.

### Policy Implications

At this time, 36 states, the District of Columbia, Guam, Puerto Rico, and the U.S. Virgin Islands have developed medical cannabis policies, and 17 states have enacted legislation to regulate adult use (68). This shift often relied on framing the issue around patients experiencing severe symptoms from terminal conditions. While an overwhelming majority of states have now enacted some medical cannabis use policy, few have specifically addressed cannabis use among EOL patients receiving nonhospice forms of palliation. Policymakers are left with 3 choices. The first option is to take a “hands-off” approach and continue allowing the states to lead and self-regulate.

The second option is for Congress to take up its regulatory role and institute legislative protections for certifying physicians and hospice care providers who allow patients to engage in authorized cannabis use, while establishing new initiatives to examine the therapeutic value for EOL patients through research. While not necessarily repealing the Controlled Substances Act, or rescheduling cannabis at the federal level, Congress could amend it to specifically allow providers to certify for state programs and fund additional research to assess clinical value for EOL patients.

The third option is to establish a comprehensive approach to ensure quality care. This includes calling on the National Institutes of Health to establish clinical guidelines for the certification of cannabis programs and instructing the Center for Medicare and Medicaid Services to establish robust training and education programs for physicians, create a specific Medicare reimbursement mechanism for patient education during cannabis certification visits, and require certified cannabis patients in the Medicare/Medicaid programs to be offered coordinated care inclusive of substance abuse prevention.

## Conclusions

This research offers needed insight into pain management approaches taken by EOL patients in the IMCP. This study is innovative in that it engages a large sample size study of cannabis users and specifically sought to explore the role cannabis plays in EOL care decision making, opioid use, and quality of life of terminal patients in palliative care. The results of the study suggest that cannabis is a viable complement to palliative care for some terminal patients. However, most of the patients in our sample use cannabis outside of formal palliative care, and some may be using it as an alternative, at least in the immediate term. It is true most EOL patients generally enroll in both palliative care and hospice care relatively late in their disease progression and this is seen as a negative quality measure (69). The findings from this study raise the question of whether access to cannabis programs further delays utilization of these services by eligible patients. Moreover, while EOL patients may also face limited access, stigma, or other factors that can independently prevent them from utilizing palliative care, it is also possible that these same factors may be the driving force in their use of cannabis. In this light, the study offers rich information for hypothesis framing and better measurement in future studies. Additional research on the intersection between cannabis use and EOL care is warranted.

## Supplementary Material

igab048_suppl_Supplementary_Material_A

igab048_suppl_Supplementary_Material_B
