# Supplementary material for: Medical Cannabis and Utilization of Nonhospice Palliative Care Services: Complements and Alternatives at End of Life
Source: Innov Aging. 2022 Jan 14;6(1):igab048. doi: 10.1093/geroni/igab048 (PMC8759444; doi:10.1093/geroni/igab048)
Supplement: igab048_suppl_Supplementary_Material_A [file igab048_suppl_supplementary_material_a.pdf]

IMCP CANNABIS AND OLDER PERSONS STUDY SURVEY

Dear Participant:

As you know, older adults can experience health issues. Often these health issues require complex and costly treatments. Responding to the treatment needs of patients, many states like Illinois have moved to legalize cannabis (marijuana) for medical use, offering a treatment alternative for a variety of medical conditions and symptoms. In Illinois, the state has allowed access to **medical cannabis** through the condition-based *Medical Cannabis Pilot Program* begun in 2016.

Because you are enrolled in the Illinois *Medical Cannabis Pilot Program*, we would like to learn more about your thoughts and experiences with cannabis and the Medical Cannabis Pilot Program (MCPP). The Illinois Department of Public Health has collaborated with researchers at the University of Illinois and the University of Iowa, to develop a research survey to collect data about your attitudes and experiences regarding cannabis as well as with the medical cannabis program. We now invite you to spend the next 15 minutes completing this survey.

If you are ready to begin or if you want to learn more about this research project before you start, please click on the link provided below. You will be taken to a secure webpage that provides information about this survey and reviews your rights as a participant in our study.

Remember we assure you there is no way for us to determine who you are and the answers you provide will not be shared with any company, government agency or anyone else not included on our research team. We explain how we protect your confidentiality in more detail prior to taking the survey.

Thank you for your consideration.

Click Here for the Survey

ONLINE CONSENT

[INSERT MOST RECENT IRB APPROVED VERSION OF ONLINE CONSENT FORM AUTHORIZING ANONYMOUS DATA COLLECTION]

ATTITUDES AND KNOWLEDGE ABOUT CANNABIS USE

These questions ask generally about your current attitudes about cannabis use and obtaining information about medical cannabis.

1. Medical cannabis use is risky.
- o Disagree
  - Some → A lot
- o Agree
  - Some → A lot
- o I don't know

2. The important people in my life have positive attitudes toward using medical cannabis.
- o Disagree
  - Some → A lot
- o Agree
  - Some → A lot
- o I don't know

3. How did you learn about ***medical cannabis***? **(*Select up to 3*)**
- o A health care provider **[/*Ask Q3a>]**
- o A friend or family member
- o A pharmacist
- o A community organization (*Senior Center, Library, Support Group, etc.*)
- o Dispensary staff
- o Visited Illinois Department of Public Health website or called IDPH directly
- o Newspaper, magazine, TV, billboards, or Internet Ads
- o Social-worker/Care-manager
- o Other: ___________________________

3a. What kind of health care provider was it? **(*Choose all that apply*)**
- o Primary care provider/My family doctor
- o Pain specialist
- o Oncologist
- o Other disease or condition specialist
- o Hospice Doctor or Nurse
- o A Home-health professional

CANNABIS USE

This next section asks about the use of cannabis.

Please keep in mind that your *answers to all questions are* ***confidential.***

4. In the **past year**, for what purpose did you use cannabis? **(*Mark all that apply*)**
- o I do not use cannabis at all **[/*Skip to Demographics – Q51>]**
- o I did not use cannabis in the past year **[/*Skip to Q14>]**
- o Medical purposes
- o Personal use (*recreational, leisure*)

5. How much did you typically pay **each month** for **cannabis**, over the past year? **(*Choose one*)**
- o Between $1 and $99
- o Between $100 and $199
- o Between $200 and $299
- o Between $300 and $399
- o More than $400

6. Thinking just about the last 30 days, on how many days did you use cannabis? _________days

7. How do you use cannabis? **(*Mark all that apply*)**
- o Smoke-inhale (*cigarette, cigar, pipe)*
- o *Vaporizer (leaf or oil)*
- o Orally by capsules (*pill or tablet*)
- o liquid tincture (*liquid dropper*)
- o Edible form (like a brownie, cookie or gummy candy)
- o Cream-ointment (*like a lotion you can rub on your skin*)

8. Where have you obtained cannabis in the past year? **(*Mark all that apply*)**
- o Cannabis Store or Dispensary
- o Someone gave or sold it to me
- o I purchased from another state
- o I ordered it from the internet

9. How frequently did you use **cannabis** in the past year? **(*Choose one*)**
- o A few times
- o 1-4 times a month
- o Once or twice per week
- o Regularly (*3 or more times per week*)
- o Daily *(1 or more times per day)*

10. How frequently did you use cannabis when you were **under 30 years old**?
- o Never
- o A few times a year
- o 1-4 times a month

- Once or twice per week
- Regularly (*3 or more times per week*)
- Daily *(1 or more times per day)*

11. How frequently did you use cannabis when you were **50 to 60 years old**?
- Never
- A few times a year
- 1-4 times a month
- Once or twice per week
- Regularly (*3 or more times per week*)
- Daily *(1 or more times per day)*

12. Personally, have you ever had any negative experiences when using cannabis?
- No **[/*Skip to Q13>]**
- Yes **[/*Skip to Q12a>]**

12a. Did this happen in the past year?
- No **[/*Ask 12b>]**
- Yes **[/*Skip to Q12c >]**

12b. Was this a long time ago (*More than 20 years*)?
- No **[/*Skip to Q13>]**
- Yes **[/*Skip to Q12c >]**

12c. Please describe: ______________________________________

13. Has anyone that you know personally (*spouse, child, sibling*) ever had any negative experiences using cannabis?
- No
- Yes
  If 'Yes', please describe: ____

CONDITIONS, SYMPTOMS, & ANTICIPATED EFFECTS

This next section asks about your ***specific conditions and symptoms*** *qualifying you for the medical cannabis program.*

Please keep in mind that your answers to all questions are ***confidential.***

14. For what medical condition were you qualified for the medical cannabis program? **(Mark all that apply)**
- ALS
- Cancer
- Glaucoma
- Multiple Sclerosis
- Parkinson's Disease
- Posttraumatic Stress Disorder (PTSD)

- Rheumatoid Arthritis
- Seizures/Epilepsy
- Severe Fibromyalgia
- Spinal Cord Disease
- Spinal Cord Injury
- Terminal illness
- Other: ____

15. [/*Skip to Demographics – Q51 if 'did not use cannabis in past year' at Q4>] For what medical ***symptoms*** do you use cannabis? **(*Mark all that apply*)**
- Pain
- Difficulty sleeping
- Emotional problems such as feeling anxious, depressed, or irritable
- Nausea, Constipation, Eating, and Digestive Issues
- Other: ____
- None of these apply to me

16. Have you completed the mail-in application to enroll in in the State's *Terminal Illness Cannabis Program*?
- No
- Yes

17. Relative to your **[/*Insert list of conditions and symptoms certified in Q14 & 15>]**, why do you use medical cannabis? **(*Mark all that apply*)**
- My prescribed medications do not help enough with my condition-symptoms
- I prefer not to take prescription medication for my condition-symptoms at all
- While I continue to take prescription medication for my condition-symptoms, I prefer not to take these prescription medications any more than necessary
- My primary doctor or specialist said cannabis would help
- My non-prescription treatments (e.g., *physical therapy, counseling*) do not help enough with my condition-symptoms
- I use as part of my palliative care
- I use as part of my hospice care
- Other:" __________________

These questions ask about your experience with the ***Illinois Medical Cannabis Program****.*

18. How did you learn about the Illinois Medical Cannabis Pilot Program (MCPP)? (***Select up to 3)***
- My health care provider **[/*Ask Q 18a Dropdown>]**
- A friend or family member
- Pharmacist
- A community organization (*Senior Center, Library, Support Group, etc.*)
- Dispensary staff
- Visited Illinois Department of Public Health website or called IDPH directly
- Newspaper, magazine, TV, billboards, or Internet Ads

- Social worker or case manager
- Other: ___________________________

18a. What kind of health care provider was it? (***Choose all that apply***)
- Primary care provider/My family doctor
- Pain specialist
- Oncologist
- Other disease or condition specialist
- Hospice Doctor or Nurse
- A Home-health professional

19. It was easy for me to find a physician who was willing to certify me for medical cannabis.
- Disagree
  Some A lot
- Agree
  Some A lot

20. The physician who certified me was one of my routine care providers.
- No **[/*Ask Q 20a>]**
- Yes **[/*Skip to 21>]**

20a. How were you able to obtain the written physician certification required to apply to the medical cannabis program?
- I had to search for a new doctor within my insurance provider network
- I had to go out of network to find a provider who would sign it
- I attended a seminar or medical cannabis event in my community that offered certification

21. Which type of physician certified your MCPP eligibility?
- A primary care provider or family doctor
- A pain specialist
- An oncologist
- Another doctor who is a specializes with treating my disease or condition
- Hospice Doctor

22. Did your health insurance cover your medical cannabis certification visit, like any other medical visit?
- No **[/*Skip to Q22a>]**
- Yes **[/*Skip to Q23>]**
- I don't know **[/*Skip to Q22a>]**

22a. Did you have to pay out-of-pocket for the certification visit?
- No
- Yes

23. My certifying physician was very knowledgeable about the medical cannabis program.

- Disagree
  Some A lot
- Agree
  Some A lot

24. How did you apply to the medical cannabis program?
- I applied directly to the program
- I attended a special certification event hosted by private group
- I went to the local health department in my city or county

25. I had someone (*like my doctor, a care provider, family member or friend*) assist me with my application
- No
- Yes

26. It was easy for me to apply to the state medical cannabis program.
- Disagree
  Some A lot
- Agree
  Some A lot

27. My approval for the MCPP came in a timely manner.
- Disagree
  Some A lot
- Agree
  Some A lot

28. Obtaining information and feedback from the MCPP is easy.
- Disagree
  Some A lot
- Agree
  Some A lot

29. How did you learn about the dispensary that provides you cannabis? **(*Choose one*)**
- The IDPH website
- A friend or family member
- Pharmacist
- Physician
- A community organization (i.e., *Senior Center, Library, Support Group, etc.*)
- Local Health Department
- Newspaper, Magazine, TV, billboards, or Internet Ads
- Social-worker/Care-manager
- Other: ___________________________________

30. It was easy for me to obtain cannabis from a licensed medical dispensary.
- Disagree

Some → A lot

- Agree

Some → A lot

31. Before you began the state program, had you been using cannabis for a medical purpose?

- No **[/*Skip to Q32>]**
- Yes

  If 'Yes', since when? (***MM/YYYY***) **[/*Skip to 31a>]**

31a. Why did you enroll in the state program and start obtaining cannabis from a dispensary? **(*Select all*)**

- Criminal danger/Illegal to obtain from other sources
- The other sources were "shady" / or unsafe
- The other sources had higher costs
- The other sources provided low-quality or inconsistent product
- The other sources lacked official oversight

OTHER MEDICATION USE

32. In the past year, have you used opioids or narcotics (*e.g., oxycodone, morphine, fentanyl, hydrocodone, tramadol*)?

- No **[/*Skip to Q33>]**
- Yes **[/*Skip to Q32a>]**

32a. Were these medications obtained with a prescription written for you?

- No
- Yes

32b. Relative to your medical cannabis use, has your current use of these medications

- Stopped
- Reduced
- No change
- Increased

33. In the past year, have you used benzodiazepines (*e.g., lorazepam, diazepam, clonazepam, Xanax*)?

- No **[/*Skip to Q34>]**
- Yes **[/*Skip to Q33a>]**

33a. Were these medications obtained without a prescription written for you?

- No
- Yes

33b. Relative to your medical cannabis use, has your current use of these medications

- Stopped
- Reduced

- No change
- Increased

34. How much do you currently pay each month for prescriptions for opioids, benzodiazepines, other non-opioid prescription pain medications, and over the counter pain medications? (***Choose one***)
- $0
- Insurance fully covers the cost of my pain medications.
- Between $1 and $99
- Between $100 and $199
- Between $200 and $299
- Between $300 and $399
- More than $400

CANNABIS AND END-OF-LIFE CARE

These questions ask about your experiences, feelings, and beliefs on the use of **cannabis for pain management at end-of-life**. This is inclusive of both ***palliative care*** and ***hospice care***.

35. How appropriate is cannabis-based pain-management for care at end-of-life?
- Not at all appropriate **[/*Skip to Demographics – Q51>]**
- Not very appropriate **[/*Skip to Demographics – Q51>]**
- Somewhat appropriate
- Very appropriate

36. *Palliative care* is special team-based supportive care, where treatment aims at controlling pain and reducing the stress of illness while you **continue to fight** a serious condition such as **cancer, ALS, Parkinson's disease, dementia, or another potentially terminal illness**. *Hospice care* is comfort care exclusively for patients who have received a **terminal prognosis of not more than 6 months** remaining. Hospice care is similar to palliation in that is aims to control pain and improve quality-of life; however, it **requires an individual to forgo treatment aimed at curing their condition**. Remember, an individual **cannot** receive both **palliative care** and **hospice care** at the same time.

Are you currently receiving palliative care for your condition?
- No **[/*Skip to Q40>]**
- Yes **[/*Skip to Q36a>]**

36a. Are you currently using cannabis specifically as part of your palliative care?
- No **[/*Skip to Q36b>]**
- Yes **[/*Skip to Q37>]**

36b. Why are you **not** including cannabis as part of your palliative care? (***Select all***)
- My palliative care team does not approve **[/*Skip to Q37a>]**
- I am worried about having cannabis around the important people in my life **[/*Skip to Q40a>]**
- I do not think that cannabis is effective for palliation **[/*Skip to Q40a>]**

- I am worried cannabis use will have a negative impact on fighting my disease **[/*Skip to Q40a>]**
- Other: ______________________________ **[/*Skip to Q40a>]**

37. Does your palliative care team approve of your medical cannabis use?
- No **[/*Skip to Q37a>]**
- Yes **[/*Skip to Q37b>]**

37a. How hesitant are you to use cannabis since your care team does not approve?
- Not at all hesitant **[/*Skip to Q38>]**
- A little hesitant **[/*Skip to Q38>]**
- Somewhat hesitant **[/*Skip to Q38>]**
- Very hesitant **[/*Skip to Q38>]**
- Extremely hesitant **[/*Skip to Q38>]**

37b. Is your provider's stance on medical cannabis part of why you selected them?
- No **[/*Skip to Q38>]**
- Yes **[/*Skip to Q38>]**

38. On a scale ranking your pain from 0-10, at what pain level do you use cannabis for your specific condition?

0-------1------2------3-----4-----5-----6-----7-----8-----9-----10
(0 = *No Pain*; 1-3 = *Mild Pain*; 4-6 = *Moderate Pain*; 7-9 = *Severe Pain*; 10 = *Worst Possible Pain*)

39. How responsive is your palliative care team to your treatment preferences?
- Not at all responsive **[/*Skip to Q40a>]**
- A little responsive **[/*Skip to Q40a>]**
- Somewhat responsive **[/*Skip to Q40a>]**
- Very responsive **[/*Skip to Q40a>]**
- Extremely responsive **[/*Skip to Q40a>]**

40. Are you currently receiving hospice care?
- No **[/*Skip to Q40a>]**
- Yes **[/*Skip to Q41>]**

40a. Have you previously received a terminal prognosis?
- No
- Yes

40b. Do you plan to enroll in hospice care when the time comes?
- No **[/*Skip to Demographics – Q51>]**
- Yes **[/*Skip to Hospice Subscale B - Q47>]**
- I don't want to think about end-of-life **[/*Skip to Demographics – Q51>]**

***Hospice Subscale* A [/*Only ask if answer 'yes' to Q40>]**

41. Where do you currently receive hospice care?
- At home
- At a residential hospice facility
- At a nursing home or assisted living facility

- o At a hospital, but through outpatient care
- o At a hospital as an admitted patient

42. Is your hospice provider a non-profit organization?
- o No
- o Yes
- o I don't know

43. Does your hospice provider approve of medical cannabis use for end-of-life patients?
- o No **[/*Skip to Q43a>]**
- o Yes **[/*Skip to Q43b>]**
- o I don't know **[/*Skip to Q44>]**

43a. How hesitant are you to use cannabis since your hospice provider does not approve?
- o Not at all hesitant **[/*Skip to Q44>]**
- o A little hesitant **[/*Skip to Q44>]**
- o Somewhat hesitant **[/*Skip to Q44>]**
- o Very hesitant **[/*Skip to Q44>]**
- o Extremely hesitant **[/*Skip to Q44>]**

43b. Is your hospice provider's stance on medical cannabis part of why you selected it?
- o No **[/*Skip to Q44>]**
- o Yes **[/*Skip to Q44>]**

44. On a scale ranking your pain from 0-10, at what pain level do you use cannabis for your specific condition?

0-------1------2------3-----4-----5-----6-----7-----8-----9-----10
(0 = *No Pain*; 1-3 = *Mild Pain*; 4-6 = *Moderate Pain*; 7-9 = *Severe Pain*; 10 = *Worst Possible Pain*)

45. How responsive is your hospice team to your treatment preferences?
- o Not at all responsive
- o A little responsive
- o Somewhat responsive
- o Very responsive
- o Extremely responsive

46. How much do you trust that your hospice team will be there at the end, when you need them most?
- o I do not trust them very much **[/*Skip to Demographics – Q51>]**
- o I trust them only a little **[/*Skip to Demographics – Q51>]**
- o I trust them somewhat **[/*Skip to Demographics – Q51>]**
- o I trust them very much **[/*Skip to Demographics – Q51>]**
- o I trust them **[/*Skip to Demographics – Q51>]**

***Hospice Subscale* B [/*Only ask if 'yes' to Q40b (ENROLL WHEN TIME COMES)>]**

47. Where do you most want to receive hospice care?
- o At home
- o At a residential hospice facility
- o At a nursing home or assisted living facility
- o At a hospital, but through outpatient care

- At a hospital as an admitted patient

48. Would you select a hospice provider specifically because they allow for cannabis use?
- No
- Yes

49. How important is a hospice team that is responsive to your advance care plans?
- Not at all important
- A little important
- Somewhat important
- Very important
- Extremely important

50. How important is having a hospice professional there at the end, when you actually make your transition?
- Not at all important **[/*Skip to Demographics – Q51>]**
- A little important **[/*Skip to Demographics – Q51>]**
- Somewhat important **[/*Skip to Demographics – Q51>]**
- Very important **[/*Skip to Demographics – Q51>]**
- Extremely important **[/*Skip to Demographics – Q51>]**

DEMOGRAPHIC AND GENERAL INFORMATION

51. County of Residence:____________________

52. YEAR of Birth
- (YYYY)

53. Sex
- Male
- Female
- Transgender

54. Are you of Hispanic, Latino, or Spanish origin?
- No, not of Hispanic, Latino, or Spanish origin.
- Yes, Mexican, Mexican American, Chicano
- Yes, Puerto Rican
- Yes, Cuban
- Yes, another Hispanic, Latino, or Spanish origin.

55. Race (***Mark all that apply***)
- White
- Black, African American, or Negro
- American Indian /Alaska Native
- Asian Indian
- Chinese
- Filipino
- Japanese
- Korean
- Vietnamese

- Other Asian
- Native Hawaiian
- Guamanian or Chamorro
- Samoan
- Other Pacific Islander
- Some other race

56. Highest degree or level of education completed
- No schooling or only kindergarten
- No high school diploma
- High School/GED
- Some college or technical school
- Associate's degree
- Bachelor's degree
- Master's degree
- Professional degree
- Doctorate degree

57. Marital Status
- Never Married
- Married
- Divorced
- Separated
- Widowed

58. Military Service
- No, never served in the U.S. military
- Yes, in the Reserves or National Guard only
- Yes, I have previously served in the U.S. Military
    - I have a VA disability rating
        - No
        - Yes
            - What percentage: ________
    - I ended my Time in Service within the past 3 years
        - No
        - Yes
    - I served in the military during the Vietnam War
        - No
        - Yes
    - I have been deployed to a military conflict on federal orders during my Time in Service
        - No
        - Yes

59. Do you have a documented disability as defined by the Americans with Disabilities Act?
- No
- Yes

60. Employment Status
- Employed for wages
- Self-employed
- Out of work for 1 year or more
- Out of work for less than a year
- Retired
- Unable to work

61. Which best describes your financial situation?
- My income covers my expenses and have extra each month
- My income covers my expenses but not much more
- I sometimes have a hard time making ends meet
- I obtain financial assistance (e.g., Medicaid, Supplemental Security Income) to help me make ends meet

HEALTH OUTCOMES & EFFECTS

62. In general, would you say your overall quality of life is:
- Poor
- Fair
- Good
- Very good
- Excellent

63. [/*Skip to Q64 if 'no use' at Q4>] How does using cannabis affect your overall quality of life?
- Make it worse
- No change
- Make it better

[If 'worse' or 'better',>] Please indicate level by dragging bar to the right.
0 → 100

64. In general, how are managing your health outcomes and expectations?
- Poor
- Fair
- Good
- Very good
- Excellent

65. [/*Skip to Q66 if 'no use' at Q4>] How does using cannabis affect your health outcomes and expectations?
- Make it worse
- No change
- Make it better

[If 'worse' or 'better',>] Please indicate level by dragging bar to the right.
0 → 100

66. In general, to what extent are you able to generally be productive? (i.e., *go to work, grocery shopping, etc.*)
- o Not at all
- o A little
- o Moderately
- o Mostly
- o Completely

67. [/*Skip to Q68 if 'no use' at Q4>] How does using cannabis affect your overall productivity?
- o Make it worse
- o No change
- o Make it better

[If 'worse' or 'better',>] Please indicate level by dragging bar to the right.
0 → 100

68. In general, how would you rate your satisfaction with your psychological wellbeing?
- o Poor
- o Fair
- o Good
- o Very good
- o Excellent

69. [/*Skip to Q70 if 'no use' at Q4>] How does using cannabis affect your psychological wellbeing?
- o Make it worse
- o No change
- o Make it better

[If 'worse' or 'better',>] Please indicate level by dragging bar to the right.
0 → 100

70. In general, how would you rate your satisfaction with your social activities and relationships?
- o Poor
- o Fair
- o Good
- o Very good
- o Excellent

71. [/*Skip to Q72 if 'no use' at Q4>] How does using cannabis affect your social activities and relationships?
- o Make it worse
- o No change
- o Make it better

[If 'worse' or 'better',>] Please indicate level by dragging bar to the right.
0 → 100

72. In general, how would you rate the frequency of your participation level in exercise and other physical activities? (i.e., dog *walking, lifting your grandkids, swimming, Zumba, Silver Sneakers program, etc.*)

- Poor
- Fair
- Good
- Very good
- Excellent

73. [/*Skip to Q74 if 'no use' at Q4>] How does using cannabis affect the frequency of your participation in exercise activities? (*i.e.,* dog *walking, lifting your grandkids, swimming, Zumba, Silver Sneakers program, etc.*)

- Make it worse
- No change
- Make it better

[If 'worse' or 'better',>] Please indicate level by dragging bar to the right.
0 → 100

74. In the past 30 days, how would you rate your pain on average?

0-------1------2------3-----4-----5-----6-----7-----8-----9-----10
(0 = *No Pain*; 1-3 = *Mild Pain*; 4-6 = *Moderate Pain*; 7-9 = *Severe Pain*; 10 = *Worst Possible Pain*)

75. [/*Skip to Q76 if 'no use' at Q4>] How does using cannabis affect your pain?

- Make it worse
- No change
- Make it better

[If 'worse' or 'better',>] Please indicate level by dragging bar to the right.
0 → 100

76. In the past 30 days, how would you rate your sleep quality?

- Very poor
- Poor
- Fair
- Good
- Very good

77. [/*Skip to Q78 if 'no use' at Q4>] How does using cannabis affect your sleep quality?

- Make it worse
- No change
- Make it better

[If 'worse' or 'better',>] Please indicate level by dragging bar to the right.
0 → 100

78. In the past 30 days, how often have you been bothered by emotional problems such as feeling anxious, depressed or irritable?

- Always

- Often
- Sometimes
- Rarely
- Never

79. **[/*Skip to Q80 if 'no use' at Q4>]** How does using cannabis affect your emotional problems?
- Make it worse
- No change
- Make it better

[If 'worse' or 'better',>] Please indicate level by dragging bar to the right.
0 100

80. In the past 30 days, how often have you had problems with nausea, constipation, eating, or other digestive issues?
- Always
- Often
- Sometimes
- Rarely
- Never

81. **[/*Skip to Q82 if 'no use' at Q4>]** How does using cannabis affect your nausea, constipation, eating, or other digestive issues?
- Make it worse
- No change
- Make it better

[If 'worse' or 'better',>] Please indicate level by dragging bar to the right.
0 100

82. In the past 30 days, have you experienced any issues with memory or thinking?
- No
- Yes

83. **[/*Skip to Q84 if 'no use' at Q4>]** How does using cannabis affect your memory or thinking?
- Make it worse
- No change
- Make it better

[If 'worse' or 'better',>] Please indicate level by dragging bar to the right.
0 100

84. In the past 12 months, have you fallen? Defined as an event when you come to rest on the ground or lower level unintentionally.
- No
- Yes

85. **[/*Skip to Q86 if 'no use' at Q4>]** How does using cannabis affect the frequency of your falls?

- Make it worse
- No change
- Make it better

[If 'worse' or 'better',>] Please indicate level by dragging bar to the right.
0 → 100

86. In the past 30 days, have you visited the Emergency Room?
- No **[/*Skip to 88>]**
- Yes **[/*Skip to 86a>]**

86a. Was this Emergency Room visit opioid related?
- No
- Yes

87. [/*Skip to Q90 if 'no use' at Q4>] How does using cannabis affect your use of the Emergency Room?
- Make it worse
- No change
- Make it better

[If 'worse' or 'better',>] Please indicate level by dragging bar to the right.
0 → 100

88. [/*Skip to 90 if 'no' to Q16 and/or 'I don't want to think about end-of-life ' on Q40b>] How has cannabis use affected end of life discussions with family and loved ones?
- Make it worse
- No change
- Make it better

[If 'worse' or 'better',>] Please indicate level by dragging bar to the right.
0 → 100

89. How has cannabis use affected your ability to manage planning for end of life, specifically?
- Make it worse
- No change
- Make it better

[If 'worse' or 'better',>] Please indicate level by dragging bar to the right.
0 → 100

90. Is there anything that we did not ask that you would like to share: **[Free Text]**

91. Would you be willing to be contacted to participate in a follow-up survey or phone interview? If so, please provide an email address where you can be reached. **[Free Text – valid email entry]**
