# Supplementary material for: Medical Cannabis and Utilization of Nonhospice Palliative Care Services: Complements and Alternatives at End of Life
Source: Innov Aging. 2022 Jan 14;6(1):igab048. doi: 10.1093/geroni/igab048 (PMC8759444; doi:10.1093/geroni/igab048)
Supplement: igab048_suppl_Supplementary_Material_B [file igab048_suppl_supplementary_material_b.docx]

Supplementary Table S1. Logistic Regression Predicting Palliative Care Utilization with Propensity Scores: Comparing Terminal Patients in Palliative Care to Terminal Patients Not Engaging Supportive Care (*n* = 572)

| Palliative Care Patients | OR | [95% CI] | p-value |
| --- | --- | --- | --- |
| *Demographics* |  |  |  |
| College degree or more | 0.45 | [0.26 – 0.78] | 0.01 |
| Married | 0.44 | [0.26– 0.73] | 0.01 |
| *Health status* |  |  |  |
| Cancer diagnosis | 2.31 | [1.35 – 3.98] | < .001 |
| Treating multiple symptoms | 0.51 | [0.30 – 0.88] | 0.02 |
| *Cannabis use and program access* |  |  |  |
| 14-day fast-track applicant | 0.42 | [0.26 – 0.67] | < .001 |

*Note*. This logistic regression included indicators for age group category, gender, race/ethnicity, prior military service, employment status, caregiver proxy use, low quality of life, frequent gastrointestinal issues, multiple symptoms, opioid use in the past year, and the propensity score as covariates.
